# Kisspeptin/Kisspeptin Receptor System in the Ovary

**DOI:** 10.3389/fendo.2017.00365

**Published:** 2018-01-04

**Authors:** Kai-Lun Hu, Hongcui Zhao, Hsun-Ming Chang, Yang Yu, Jie Qiao

**Affiliations:** ^1^Beijing Key Laboratory of Reproductive Endocrinology and Assisted Reproductive Technology and Key Laboratory of Assisted Reproduction, Ministry of Education, Center for Reproductive Medicine, Department of Obstetrics and Gynecology, Peking University Third Hospital, Beijing, China

**Keywords:** kisspeptin, kisspeptin receptor, ovarian function, follicular development, oocyte maturation, ovulation, steroidogenesis, kisspeptin signaling

## Abstract

Kisspeptins are a family of neuropeptides that are critical for initiating puberty and regulating ovulation in sexually mature females *via* the central control of the hypothalamic–pituitary–gonadal axis. Recent studies have shown that kisspeptin and its receptor kisspeptin receptor (KISS1R) are expressed in the mammalian ovary. Convincing evidence indicates that kisspeptins can activate a wide variety of signals *via* its binding to KISS1R. Experimental data gathered recently suggest a putative role of kisspeptin signaling in the direct control of ovarian function, including follicular development, oocyte maturation, steroidogenesis, and ovulation. Dysregulation or naturally occurring mutations of the kisspeptin/KISS1R system may negatively affect the ovarian function, leading to reproductive pathology or female infertility. A comprehensive understanding of the expression, actions, and underlying molecular mechanisms of this system in the human ovary is essential for novel approaches to therapeutic and diagnostic interventions in reproductive diseases and infertility.

## Introduction

Female reproduction is a highly orchestrated and regulated process controlled by the hypothalamic–pituitary–ovarian (HPO) axis. The pulsatile gonadotropin-releasing hormone (GnRH), and therefore gonadotropins (FSH and LH), secretion primarily governs the HPO axis at puberty and maintains the cyclic function in adulthood (1). This tonic GnRH/gonadotropins secretion is modulated by a negative feedback effect of serum estrogen secreted from the growing ovarian follicles (2). Apart from the pulsatile secretion of GnRH, the surge mode of GnRH release is characterized by a large amount of LH secretion, which is required for triggering ovulation in female mammals (3). During the periovulatory stage, the high serum level of estrogen exerts its positive feedback influence upon GnRH neurons to induce a GnRH surge and hence the LH surge (4). Studies related to the underlying cellular and molecular mechanisms of the negative and positive feedback effects of estrogen have been of considerable interest. Even though the critical role of GnRH in regulating female reproduction, there exist several functional limitations of the GnRH neuronal network. The major issue is that GnRH neurons do not express estrogen receptor α, the principle receptor that mediates both negative and positive estrogen feedback actions (5).

In the past decade, emerging studies have found that kisspeptin (KISS1) is the upstream regulator of pulsatile and surge GnRH release, with indispensable roles in female reproduction, including gonadotropin secretion, puberty onset, brain sex differentiation, ovulation, and metabolic regulation of fertility (6, 7). In mammals, two populations of hypothalamus kisspeptin neurons, anteroventral periventricular nucleus (AVPV) and arcuate nucleus (ARC), have been identified to play different functional roles in exerting the positive and negative feedback actions in response to estrogen (8, 9). Specifically, kisspeptins function through a G-protein-coupled receptor, kisspeptin receptor (KISS1R) to stimulate the release of GnRH (and subsequent secretion of FSH and LH) in many mammals. Notably, in humans and mice, inactivating mutations in either kisspeptin or KISS1R lead to the phenotype of hypogonadotropic hypogonadism. In addition, emerging evidence indicates the potential physiological roles of extra-hypothalamic kisspeptins in modulating the activity of diverse systems in the brain and many peripheral organs (10–14). Several studies have demonstrated that kisspeptins and their putative receptor, KISS1R are expressed across different types of tissues (15–19). Therefore, kisspeptins may exert their direct actions on various types of tissues in an autocrine/paracrine manner depending on different physiological conditions. With regard to the reproductive function, an increasing number of reports have shown that the reproductive tissues, such as ovary, female genital tract, placenta, and testis, can express functional form of kisspeptin/KISSR system among various species including humans (18, 20, 21). Furthermore, several publications have appeared in recent years documenting that locally produced kisspeptin/KISSR directly participates in a series of physiological and pathological activities in the ovary. Indeed, the extra-hypothalamic roles of kisspeptins have recently attracted special attention in fields related to reproductive biology and clinical reproductive medicine. This review will focus mainly on available literature related to the pathophysiological roles of kisspeptin/KISSR in regulating ovarian function and summarize our current understanding of the mechanisms by which kisspeptin exert its cellular actions as well as the therapeutic implications of kisspeptins in reproductive medicine.

## Methods

A systematic literature search was performed using PubMed and Web of Science for all English-language articles up to November 2017. A systematic review of English-language publications was carried out using the following keywords: Kiss1, kisspeptin, metastin, KISS1R, GPR54, ovary, kisspeptin signaling, premature ovarian failure, PCOS, endometriosis, follicular development, steroidogenesis, oocyte maturation, ovulation, knockout, and therapeutic application. The goal of this review is to summarize the latest studies regarding the direct roles and physiological significance of kisspeptin/KISS1R in the ovary and discuss some molecular mechanisms and potential therapeutic targets in reproductive diseases.

## Discovery of Kisspeptin and KISS1R

**Figure 1:**
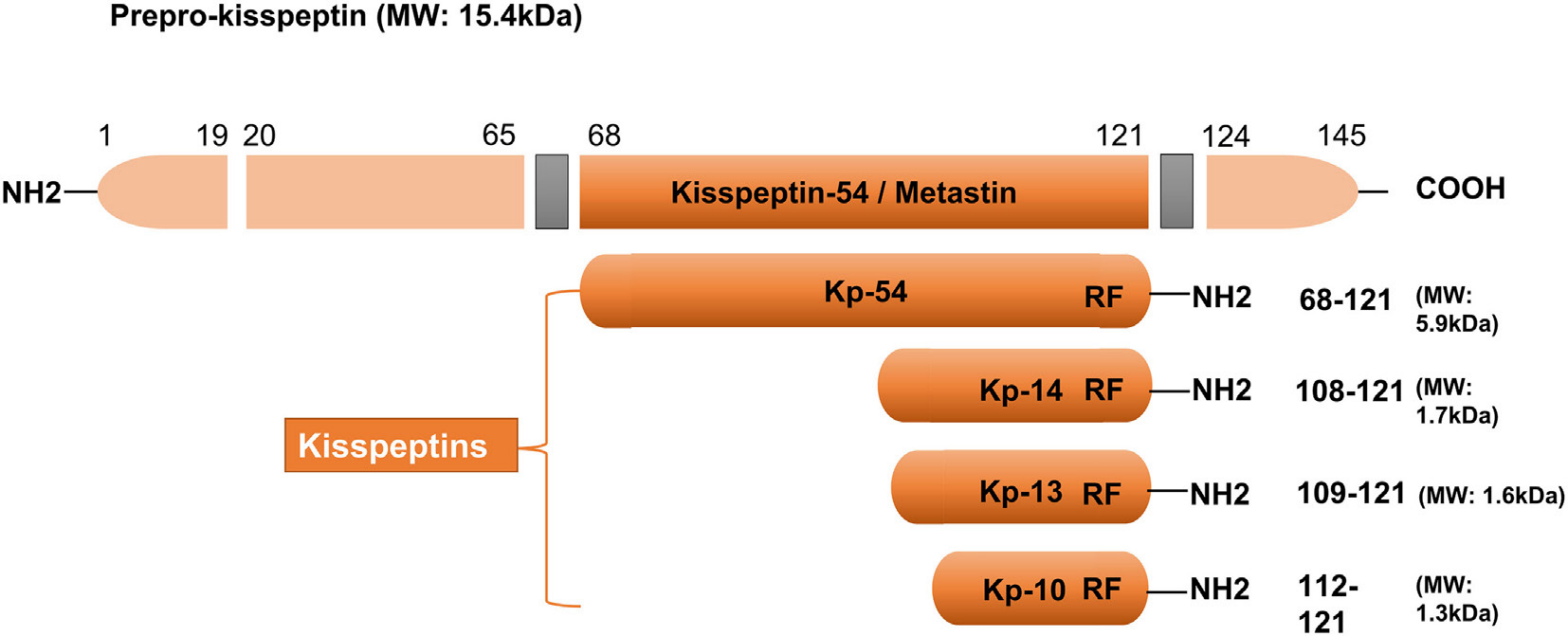
Major structural features of human kisspeptins, the products of the *Kiss1* gene. Different kisspeptins are generated by the cleavage from a common precursor, the prepro-kisspeptin. The prepro-kisspeptin contains 145 amino acids, with a 19-amino acid signal peptide and a central 54-amino acid region, kisspeptin-54 (Kp-54; formerly termed as metastin). Further cleavage of metastin generates kisspeptins of lower molecular weight: kisspeptin-14 (Kp-14), Kp-13, and Kp-10. All kisspeptins contain the RF-amide motif that is able to bind and activate kisspeptin receptor. Modified from Ref. (6).

In 1996, kisspeptin (the 145 amino acid) and its encoding gene *KISS1* were first identified as a suppressor (and a gene) of human malignant melanoma in Hershey, Pennsylvania, USA—the hometown of the famous Hershey’s kisses chocolates (22). The name of KISS1 was derived from these sweets, with the “SS” representative of “suppressor sequence.” In human, *KISS1* is located on the long (q) arm of chromosome 1 at q32. *KISS1* encodes an unstable and biologically inactive intermediate prepropeptide of 145 amino acids, which is further post-translationally converted to four biologically active peptides distinguished on the basis of their number of amino acid: kisspeptin-54, 14, 13, and 10 (Figure 1). All of the peptides have a C-terminal region that contains an Arg–Phe–NH_2_ motif characteristic of the RF-amide peptide family, which allows them to fully activate KISS1R. Based on structural similarities and their common origin as *KISS1*-derived peptides, the term kisspeptin was globally used to define this family (6, 23). Kisspeptin-54 was initially termed as “metastin” because of its capacity to inhibit tumor metastasis. This peptide has been considered as the major product of the human *KISS1* gene (19). Whereas in rats and mice, the largest proteolytic product of the kisspeptin precursor is kisspeptin-52 (composed of 52 amino acids), and the terminal RF-amide signature is substituted by an Arg–Tyr–NH_2_ motif (6). Kisspeptin-54, -14, and -13 as well as a shorter peptide designated kisspeptin-10 have the same affinity and efficacy on KISS1R in both humans and rats, indicating that the C-terminal part of the peptides is responsible for the high-affinity binding and the activation of KISS1R (23).

Kisspeptin receptor is a seven-transmembrane G-protein-coupled receptor, which was firstly identified in the rat brain as an orphan receptor with around 40% sequence similarity with the transmembrane region of galanin receptors (24). Subsequently, the human ortholog of KISS1R was cloned and cataloged as a putative receptor for KISS1-derived peptides (19, 25). Since various groups of researchers independently noted its presence or studied its physiological roles, KISS1R has been given various different names, including KISS1R, GPR54, AXOR12, hOT7T175, CPPB1, and HH8 (19, 23–25). It was not until 2003 that the physiological role of kisspeptins and their receptor, KISS1R in the neuroendocrine–reproductive axis was identified (26, 27), which thereafter revolutionized the field of reproductive physiology. These findings suggest that kisspeptins and their receptor KISS1R play a critical role as gatekeepers of sexual maturation during puberty onset and central processors for the dynamic regulation of the gonadotropic axis at adulthood.

## The Kisspeptin/KISS1R System in the Ovary

The action of kisspeptin/KISS1R in the ovary presupposes the presence of functional KISS1R and its endogenous ligand kisspeptin. Expression of both kisspeptin and KISS1R has been reported in a variety of tissues, including adipose tissue, pancreas, liver, small intestine, peripheral blood lymphocytes, testis, lymph nodes, aorta, coronary artery, and umbilical vein, female tract, and particularly abundant in placenta and the central nervous system (12, 21, 28). Terao et al. first reported that kisspeptin mRNA was prominently expressed in the rat ovary, suggesting a locally functional role of kisspeptin in this reproductive tissue (12). Specific structure or cell expression of kisspeptin and its receptor KISS1R had not been indicated until 2006, when Castellano et al. found that kisspeptin and KISS1R were both expressed in theca cells, corpora lutea, and interstitial tissues (17). While there are inconsistent results from later studies that are particular puzzling with regard to the cell expression of Kiss1/KISS1R even in the same species (14, 20, 29–36) (summarized in Table 1). For example, some studies demonstrated the absence of kisspeptin/KISS1R in granulosa cells (GCs) of rat ovary (17, 30), whereas other studies indicated the strong expression of kisspeptin or KISS1R in rat GCs (14, 37, 38). One study has identified that GC was the major site for kisspeptin synthesis in rats, as *Kiss1* mRNA expression was significantly higher in the GCs compared with the theca cells and other ovarian cells (38). However, other studies showed that *KISS1R* mRNA was equally distributed between the GCs and other cells of the residual ovary. These inconsistencies in the expression of the kisspeptin/KISS1R system in the ovary are partly because of the different methods employed to examine their presence. In addition, the discrepancies of age and ovarian tissues and cells obtained from different estrous/menstrual cycles can significantly affect the expression patterns (17, 20, 32–35, 38–41). Similar to other species, the follicular expression of kisspeptin/KISS1R is gradually increased as the follicles grow, with a highest level at the preovulatory stage in humans (20, 35, 36), which is partly due to the stimulatory effect of the gradually increased gonadotropins on the expression of kisspeptin at this stage (17). In gene knockout models, *Kiss1*^−/−^ or *Kiss1R*^−/−^ mice showed small ovarian size and weight compared to the wild-type counterparts (see Table 2). Initially, no such difference in ovarian weight was detected between wild-type and *Kiss1R*^−/−^ females in 3-week-old animals. Eventually, in 9-week-old and 7-month-old animals, there was a significant decrease in reproductive organ weights in the female *Kiss1R*^−/−^ mice (42). These findings suggest a possible age-related physiological role for the kisspeptin/KISS1R system in ovarian physiology and that this system functions mainly at the puberty and adult stages.

**Table 1:** Expression of the kisspeptin/kisspeptin receptor system in the ovary.

Reference	Kisspeptins	GPR54	Methods	Species
Garcia-Ortega et al. (29)	MGCs, CCs	MGCs, CCs	RT-qPCR	Human
Laoharatchatathanin et al. (14)	GCs	GCs, interstitial cells	RT-qPCR	Rat
Zhou et al. (30)	TCs, oocyte, interstitial cells	TCs	IH	Rat
Garcia-Ortega et al. (31)	MGCs, CCs	MGCs, CCs	RT-qPCR, IF, WB	Human
Merhi et al. (32)	MGCs (−) CCs (−)	CCs	RT-qPCR	Human
Merhi et al. (32)	Ovary	Ovary	RT-qPCR	Mouse
Cielesh et al. (33)	Oocyte, CL, GCs, TCs (−)	Oocyte, CL, GCs, TCs (−)	IH	Canine
Gaytan et al. (41)	TCs, CL, interstitial and epithelium cells; GCs (−), SCs (−)	TCs, SCs, GLCs; TLCs (−)	RT-qPCR, IH	Human, marmoset
Castellano et al. (17)	GCs (−), TCs, TCLs, GLCs, CL, oocyte, interstitial	GCs (−), TCs, CL, interstitial; SCs (−)	RT-qPCR, IH	Rat
Hsu et al. (34)	Oocyte, GCs, TCs	GCs (−), oocyte (−), interstitial	IH	Mouse
Shahed and Young (20)	CL, GCs, TCs, interstitial (−)	CL, GCs, TCs (−)	RT-qPCR, IH	Hamster
Peng et al. (37)	TCs, CL, interstitial, GC (−)	CL, GC, interstitial (−)	RT-qPCR, IH	Rat
Xiao et al. (43)	CL, GCs	CL	RT-qPCR, IF	Chicken
Saadeldin et al. (44)	CCs (−), oocyte, FF	CCs, oocyte	RT-qPCR, IF, EIA	Pig
Mondal et al. (35)	AF, CL	No data	EIA	Cow
Mondal et al. (36)	FF	No data	EIA	Cow
Terao et al. (12)	Ovary	No data	RT-qPCR	Rat
Fernandois et al. (40)	Ovary	No data	RT-qPCR, WB	Rat
Fernandois et al. (39)	Ovary	No data	RT-qPCR, WB	Rat
Dorfman et al. (45)	GCs	Oocyte	RT-qPCR, IH	Mouse
Cejudo Roman et al. (21)	Ovary	Ovary	RT-qPCR,	Human
Ricu et al. (38)	CL, interstitial gland, TCs, GCs	Ovary, GCs	RT-qPCR, IH, IF	Rat

*AF, antral follicle; CL, corpus luteum; CCs, cumulus cells; GCs, granulosa cells; FF, follicular fluid; TCs, theca cells; MGCs, mural granulosa cells; GLCs, granulosa-lutein cells; TLCs, theca-lutein cells; SCs, stromal cells; EIA, enzyme immunoassay; IF, immunofluorescence; IH, immunohistochemistry; WB, western blot; RT-qPCR, quantitative real-time PCR*.

**Table 2:** Summary of the ovarian effects of *Kiss1/Kiss1r* in genetically modified or mutant models.

Reference	Mutant model	Fertility	Ovarian effects	Serum Gn	Species
Funes et al. (46)	*Kiss1r*^−/−^	Infertile	Ovary size↓	No data	Mouse
Seminara et al. (27)	*Kiss1r*^+/−^	Fertile	Normal	No data	Mouse
Seminara et al. (27)	*Kiss1r*^−/−^	Induced ovulation when injection of pregnant mare serum gonadotropin and HCG, but no data as to fertility	Ovary weight↓; primary and secondary follicles and occasionally an early antral follicle, but no large graafian follicles or corpus luteum (CL)	FSH↓ LH↓	Mouse
Colledge (47)	*Kiss1r*^−/−^	No data	Ovary weight↓	FSH↓ LH↓	Mouse
Colledge (47)	*Kiss1*^−/−^	No data	Ovary weight↓	FSH↓ LH↓	Mouse
Chan et al. (48)	*Kiss1*^−/−^	No data	Follicles at all stages of development and large numbers of atretic follicles, no CL	FSH↓ LH (−)	Mouse
Uenoyama et al. (49)	*Kiss1*^−/−^	No data	Ovary size↓	FSH↓ LH↓	Rat
Kauffman et al. (50)	*Kiss1r*^−/−^	No data	Ovary size↓	No data	Mouse
Mayer and Boehm (51)	Specific *Kiss1* KO in neurons	Fertile	Ovary weight↓; all stages of follicles and CL	LH (−)	Mouse
Mayer and Boehm (51)	Specific *Kiss1r* KO in neurons	Fertile	Ovary weight↓; All stages of follicles and CL	LH↓ (insignificant)	Mouse
Gaytan et al. (52)	*Kiss1r*^+/−^	Fertile before 48 weeks old; infertile at 48 weeks old	Ovulated oocyte↓, primary follicle↓ (by 16 weeks of age); premature ovarian failure (after 32 weeks of age)	FSH (−) LH (−) before 48 weeks old; FSH↑ LH (−) at 48 weeks old	Mouse
Gaytan et al. (52)	*Kiss1r*^−/−^	No data	Ovary size↓; primary, and secondary follicles; all early-antral follicles showed signs of atresia; no large antral follicles or corpora lutea	FSH↓ LH↓	Mouse
Gaytan et al. (53)	*Kiss1r*^−/−^	No data	Growing follicles↓, resting follicles↑	No data	Mouse
Garcia-Galiano et al. (42)	*Kiss1r*^−/−^	No data	Ovary weight (−) (by 3 weeks of age); ovary weight↓ (by 9 weeks of age); all stage follicles; atretic follicles↑, CL↓	No data	Mouse
Lapatto et al. (54)	*Kiss1r*^+/−^	Fertile	Ovary weight (−)	FSH (−) LH (−)	Mouse
Lapatto et al. (54)	*Kiss1r*^−/−^	Infertile	Ovary weight↓; absence of preovulatory follicles and CL, atretic follicles↑	FSH↓ LH (−)	Mouse
Lapatto et al. (54)	*Kiss1*^+/−^	Fertile	Ovary weight (−); all stages of follicles and CL, atretic follicles↑	FSH (−) LH↑	Mouse
Lapatto et al. (54)	*Kiss1*^−/−^	Infertile	Ovary weight↓/t (−); absence of preovulatory follicles and CL, atretic follicles↑, presence of multiple large cyst	FSH↓ LH (−)	Mouse
d’Anglemont de Tassigny et al. (55)	*Kiss1*^−/−^	No data	Ovary size↓; absence of preovulatory follicles and CL, atretic follicles↑	FSH↓ LH↓	Mouse
Kirilov et al. (56)	Specific *Kiss1r* KO in neurons	Infertile	Ovary size↓, absence of CL, atretic follicles↑	FSH↓ LH (−)	Mouse

## Roles of the Kisspeptin/KISS1R System in Regulating Ovarian Function

### Follicular Development

The population of primordial follicles decreases at variable rates until menopause in humans and infertility in rodents. During follicular development, the selection and activation of primordial follicles in the ovarian pool (ovarian reserve), established early in life, provides all growing follicles, including primary, secondary, small antral, and large antral follicles, and ovulated oocytes.

Using an implanted mini-osmotic pump containing kisspeptin or kisspeptin antagonist P234 for 28 days, Fernandois et al. evaluate the long-term effect of kisspeptin on ovarian follicular development (39). In 6- and 10-month-old rats, ovaries infused with a low dose of kisspeptin had a fewer number of antral follicles, but an increased number of preovulatory follicles and corpora lutea. On the contrary, ovaries infused with P234 had an increased number of antral follicles and a decreased number of preovulatory follicles and corpora lutea. This study also showed that kisspeptin attenuated the initial follicle recruitment (primary to secondary) by downregulating the expression of FSH receptor (FSHR). Apart from the downregulation of FSHR, kisspeptin can suppress the initial follicle recruitment through the upregulation of circulating anti-Müllerian hormone (AMH). AMH is primarily secreted by the secondary and small antral follicles and is a biomarker for ovarian reserve (57) due to its ability to inhibit the activation of primordial follicles (58). Interestingly, Fernandois et al. found that local administration of kisspeptin increased plasma AMH, whereas administration of P234 decreased plasma AMH in 6- and 10-month-old rats (39). Therefore, kisspeptin may negatively regulate preantral (including primordial, primary, and secondary follicles in this review) follicular development through upregulating AMH and downregulating FSHR expression in the ovary. However, there was an intriguing phenomenon that kisspeptin can induce an accumulation of preovulatory follicles and a decrease in the number of small antral follicles (39). We may speculate that kisspeptin inhibits the growth of preantral and small antral follicles by negatively regulating the expression of FSHR. On the other hand, kisspeptin upregulates AMH expression and promotes the maturation of large antral follicles, as the same phenotype shown in *Kiss1*^−/−^ or *Kiss1r*^−/−^ knockout models (54, 55). Intriguingly, low dose of LH has been shown to suppress the development of small ovarian follicles and stimulate the growth of large ovarian follicles (59–64). Collectively, these findings indicate that kisspeptin could be the downstream target of LH signals to regulate the follicular development. In line with these findings, Castellano and coworkers found that Kiss1 mRNA was significantly increased in rat ovary following the injection of hCG (17).

In gene knockout models, both *Kiss1r*^−/−^ and *Kiss1*^−/−^ mice display significantly reduced ovarian weight and size (46, 47, 49, 50), which may be resulted from the absence of large follicles (Table 2). Interestingly, the *Kiss1r* haploinsufficient (*Kiss1r*^+/−^) mice have significantly decreased ovarian kiss1r expression and exhibit premature ovarian failure (POF) at 32-week-old age, with a substantial loss of preantral follicles and increased percentage of atretic follicles. The depletion of these follicles seems not because of the defect in gonadotropin secretion as the exhaustion of follicular reserve cannot be rescued by gonadotropin replacement (52). Moreover, the gonadotropin levels were not significantly different between the wild-type and *Kiss1r*^+/−^ mice as demonstrated in another study (54). Interestingly, the discrepancy of the number of preantral and antral follicles between *Kiss1r*^+/−^ and wild-type mice is related to age. Indeed, no significant differences in preantral follicle development were observed between wild-type and Kiss1r^+/−^ mice before puberty, but the progressively decreased number of preantral follicles were detected in Kiss1r haploinsufficient mice after puberty, and thereafter until the age of 32-week olds. Consistent with these results, immature ovaries showed low to negligible levels of Kiss1 mRNA, which were significantly enhanced by gonadotropin priming (17). Taken together, all these findings strongly suggest that functional role of kisspeptin in regulating follicular development mainly occurs after puberty, which is in consistence with the age-related expression of kisspeptin in the ovary (17, 32, 39).

Increased atretic follicles were also observed in the ovaries obtained from *KISS1R*^−/−^ humans and *Kiss1*^−/−^ mice (48, 54, 55). However, the increased number of atretic follicles cannot be totally attributed to the local kisspeptin/KISS1R signals, as the decreased FSH levels caused by the neural *Kiss1/Kiss1r* knockout and subsequently the downregulation of hypothalamic–pituitary–gonadal axis can also induce the atretic changes of the follicles (56, 65) (Table 2). Nevertheless, one study using ovarian histology showed the presence of all stages of follicular development and corpora lutea in mice with targeted ablation of *Kiss1* and/or *Kiss1r* expressing neurons (reduced more than 90%). Interestingly, the LH levels of mutant mice were lower than the wild-type mice, without showing the FSH levels (51). These results suggested the indispensable role of the neural kisspeptin/KISS1R system in regulating follicular development. Future studies aimed at addressing the functional role of intraovarian kisspeptin/KISSR1 system in regulating follicular development using the established ovary-specific *Kiss1/Kiss1r* knockout model will be urgently required.

## Oocyte Maturation

Evidence from two independent studies showed that kisspeptin-54 injection could trigger human oocyte maturation effectively and safely (66, 67). Given that kisspeptin-54 is able to cross the blood–brain barrier and then stimulates LH release to the peripheral circulation (44), we can expect a dramatically elevated level of LH in plasma following the kisspeptin-54 injection as reported in two studies (66, 67). Therefore, it is difficult to establish a theoretical concept that kisspeptin can directly induce oocyte maturation in the ovary because oocyte maturation can also be induced by the elevated LH level. Nevertheless, kisspeptin has been shown to enhance *in vitro* maturation of the oocytes of pigs (44) and sheep (68). However, these studies used an *in vitro* culture system of the cumulus–oophorus complex instead of the denuded oocytes. Since both cumulus cells (CCs) and oocyte can express KISS1R in pigs and mice (44, 45), it is easily confused whether the kisspeptin-induced oocyte maturation is mediated by the CCs or the oocyte itself. One interesting result derived from these studies is that during *in vitro* maturation of pig cumulus–oophorus complex, treatment with kisspeptin-10 resulted in temporally elevated expression of GDF9 and BMP15 in oocytes, two essential growth factors involved in regulating folliculogenesis, ovulation, luteinization, oocyte maturation, and developmental competency (69–71). Furthermore, these effects are potentially mediated *via* a MAPK signaling pathway in GCs (72, 73). On the other hand, administration of kisspeptin-10 also upregulated the expression of C-MOS in oocytes, which plays a crucial role in promoting the meiosis process, formation of normal spindle and chromosome, and reactivation of purified maturation promoting factor after first meiosis (74–77). Collectively, these findings suggest that kisspeptin-induced oocyte maturation is mediated by the upregulation of C-MOS, GDF9, and BMP15. To the best of our knowledge, the utilization of kisspeptin to induce *in vitro* maturation of human oocytes has not been reported.

## Ovulation

Peripheral administration of kisspeptin has been reported to induce ovulation in rats (78) and ewes (79). Since plasma levels of gonadotropins (FSH and LH) significantly increased after the administration of kisspeptin, the stimulatory effect of kisspeptin on ovulation is most likely at the hypothalamus instead of at the ovary.

In gene knockout mice, depletion of *Kiss1r* can induce ovulation after standard gonadotropin priming (27), suggesting that the ovarian kisspeptin signaling is not mandatory for ovulation. In addition, in *Kiss1r*^−/−^ female mice following the extended GnRH plus gonadotropin stimulation, newly formed corpora lutea could be observed in the ovaries, and cumulus–oophorus complexes could be found in the oviducts (52). Although there seems no significant difference of oocyte quality between wild-type and *Kiss1r*^−/−^ female mice, null animals presented significantly fewer ovulated oocytes and corpora lutea (52), suggesting that the GnRH plus gonadotropin stimulation is not enough to reverse the functional loss due to *Kiss1r* knockout. Given that ovaries from nearly all *Kiss1r*^−/−^ and many *Kiss1*^−/−^ mice do not contain follicles past the antral follicle stage (54), and most *Kiss1*^−/−^ mice and at least one *Kiss1r*^−/−^ mouse exhibit multiple large cysts with no sign of ovulation, the depletion of kisspeptin/KISS1R system significantly influences the process of ovulation. In neuron-specific *Kiss1* and *Kiss1r* knockout mice, the ovarian histology showed follicles at all developmental stages and the presence of corpora lutea. All female mutant mice can produce offspring when mated to wild-type males (51). These two independent studies strongly suggest that the locally produced kisspeptin/KISS1R system, instead of that in the neuron, may be involved in the process of ovulation and oocyte maturation. However, these findings cannot completely exclude the central role of the neural kisspeptin/KISS1R system in controlling the LH surge and the subsequent ovulation.

COX-2/prostaglandins have a crucial role in the ovulatory process (80–82). Gaytan et al. found that inhibition of COX-2 in cyclic female rats resulted in a dramatic drop of ovarian *Kiss1* mRNA levels at the time of ovulation, which was fully rescued by the coadministration of prostaglandin E2 (41). In addition, injection of hCG increased the *Kiss1* mRNA level in the ovary, which was completely reversed by the inhibition of COX-2 (41). These results indicate that kisspeptin is one of the downstream targets of COX-2/prostaglandins, and that kisspeptin may participate in the process of ovulation. In rat ovaries, the *Kiss1* mRNA levels fluctuated in a cyclic-dependent manner, with a robust increase shortly before ovulation, suggesting a functional role of kisspeptin at the time of ovulation (17). In line with this result, pregnant mare serum gonadotropin evoked a significant increase in ovarian *Kiss1* mRNA level that was further enhanced by the injection of an ovulatory dose of hCG. Furthermore, the rise of the ovarian *Kiss1* mRNA level was prevented by the blockade of LH surge using an antagonist of GnRH (17). In both 6- and 10-month-old rats, ovarian infusion with kisspeptin increased the number of corpora lutea, while infusion with P234 decreased the number of corpora lutea (39), suggesting that locally increased kisspeptin may promote the process of ovulation. Interestingly, an exposure of female rats to the high-fat diets resulted in a downregulation of ovarian *Kiss1* mRNA and kisspeptins, which is likely associated with the obesity-related ovulatory dysfunction (30).

## Steroidogenesis

The role of kisspeptins in the regulation of endocrine system was first identified in 2001 showing that kisspeptin was highly expressed in human placenta, pituitary gland, pancreas, and spinal cord and was an endogenous stimulator of oxytocin (23). In rat ovaries, kisspeptin was intensively expressed in morphologically discernible steroidogenic luteal cells of newly formed copora lutea (17). Two reports documented the direct stimulatory effects of kisspeptin on the secretion of progesterone in chicken GCs (43) and rat luteal cells (37), respectively. The synthesis of progesterone is controlled by a series of processing enzymes, including steroidogenic acute regulatory protein (StAR), cytochrome P_450_ side-chain cleavage (P450scc) enzyme, and 3β-hydroxysteroid dehydrogenase (3β-HSD) enzyme (83). The mRNA levels of all these progesterone-producing enzymes in chicken GCs were significantly increased when treated with kisspeptin-10 (43). In rat luteal cells, treatment with kisspeptin alone had no significant effect on 3β-HSD mRNA level, while cotreatment with kisspeptin and hCG significantly increased the transcript level of 3β-HSD (37). In addition, treatment with kisspeptin alone increased the mRNA levels of StAR and CYP11A, and these stimulatory effects were enhanced when cotreated with hCG (37). In line with these results, hCG stimulated the expression of kisspeptin in rat GCs, and the hCG-induced increase in progesterone production was suppressed by a kisspeptin antagonist P234 (14), suggesting an indispensable role of ovarian kisspeptin in the regulation of progesterone production.

Unlike the stimulatory effect on progesterone production, kisspeptin has no effect on estrogen production in rat luteal cells (37). However, whether kisspeptin can promote the estrogen synthesis in GCs of the growing follicle during the mid- and late-proliferative phase, when the expression of kisspeptin reaches peak levels, has not been investigated. Neurokinin B (NKB) stimulate kisspeptin secretion in an autocrine and/or paracrine manner in neurons [reviewed in Skorupskaite et al. (84)]. Interestingly, NKB and its receptor were coexpressed with kisspeptin and KISS1R in human GCs and CCs (14, 29, 31). In addition, a recent study showed that NKB exerted a direct effect on stimulating estradiol production in zebrafish follicular cells and human GCs *via* the activation of ERK signaling (85). It is likely that kisspeptin/KISS1R system is involved in NKB-induced estrogen production, as kisspeptin acts as a downstream mediator of NKB in the hypothalamus and that kisspeptin can stimulate the activation of ERK signaling (discussed later). Further studies are required to confirm the functional role of intraovarian kisspeptin in regulating steroidogenesis in human GCs of growing follicles.

## The Kisspeptin Signaling Pathway

**Figure 2:**
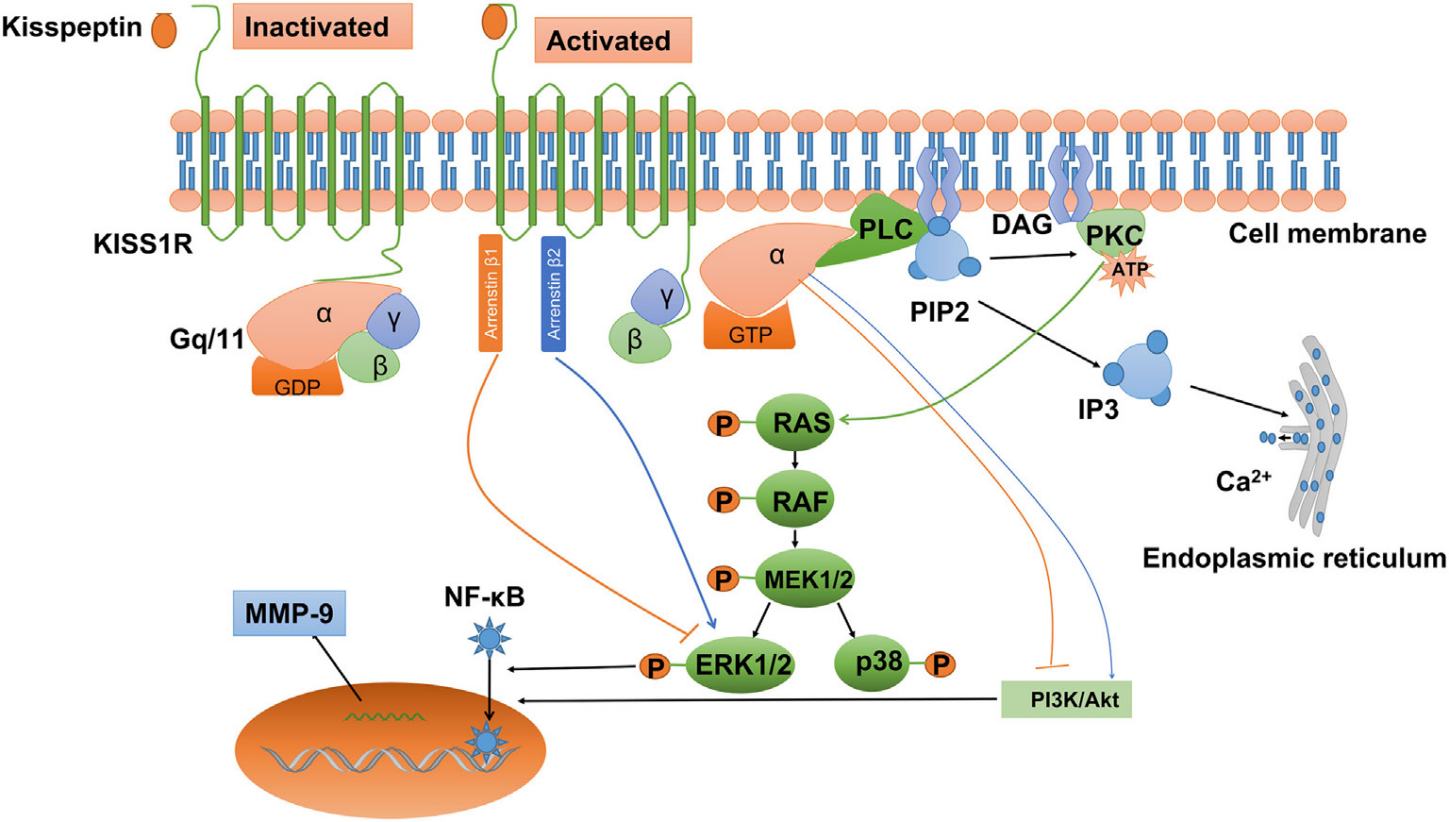
Kisspeptin/kisspeptin receptor (KISS1R) signaling at a glance. KISS1R is a seven-transmembrane domain, Gq/11-coupled receptor. Upon binding of kisspeptin, the intracellular portion of KISS1R phosphorylates Gq/11. The α-subunit of Gq/11 activates PLC, which cleaves PIP2 into IP3 and DAG. IP3 promotes intracellular Ca^2+^ release from the endoplasmic reticulum, while DAG activates a signaling cascade by phosphorylating PKC. PKC activation induces the phosphorylation of MAP kinases, such as ERK1/2 and p38. In addition, activation of KISS1R recruits arrestin-1 and -2, which downregulated and upregulated phosphorylated ERK1/2 levels, respectively. The activation of KISS1R can stimulate or inhibit the phosphorylation of PI3K/Akt, depending on the cell types, but the intermediator is not investigated. Activated KISS1R also enhances the expression of MMP-9 *via* PI3K/Akt/NF-κB or ERK/NF-κB signaling. DAG, diacylglycerol; ERK1/2, extracellular signal-regulated kinase; IP3, inositol 1,4,5-triphosphate; PI3K, phosphatidylinositol-3-kinase; MMP-9, matrix metalloproteinase-9; NF-κB, nuclear factor κB; PIP2, phosphatidylinositol 4,5-bisphosphate; PKC, protein kinase C; PLC, phospholipase C.

Convincing evidence indicates that kisspeptin can activate a wide variety of signals *via* KISS1R. Being a G-protein-coupled receptor, KISS1R belongs to the subgroup of typical Gq/11 protein-associated receptors. After kisspeptin binds to KISS1R, the phosphorylated Gq/11 protein activates phospholipase C (PLC)-β, which leads to the activation of various second messengers, including phosphatidylinositol 4,5-bisphosphate (PIP2) hydrolysis, accumulation of inositol-(1,4,5)-triphosphate (IP3), and diacylglycerol, protein kinase C (PKC) activation, and intracellular Ca^2+^ mobilization and release (Figure 2) (19, 23, 25, 86, 87). It has been reported that activation of PKC is required for LH-induced progesterone synthesis the preovulatory follicles of rats, hen, pigs, and quails (88–92). In addition, activation of PKC activity is required to promote the ERK signaling cascade that ultimately facilitates LH-induced progesterone production (93). Consistent with this result, Peng et al. showed that kisspeptin promoted progesterone synthesis by phosphorylating ERK1/2 (37). Therefore, the PLC–PKC–ERK signaling pathway most likely mediates progesterone synthesis in granulosa-luteal cells.

Ca^2+^ is initially stored in the lumen of the endoplasmic reticulum. Following the signal from IP3, Ca^2+^ is released through specialized channels, and therefore the free Ca^2+^ concentration in the cytoplasm is elevated (94). LH induces a rapid rise of intracellular Ca^2+^ that is released from Ca^2+^ stores in the cumulus layers, and subsequently an increased Ca^2+^ efflux into the oocyte (95). The increase of Ca^2+^ in the oocyte is thought to play a role in controlling either spontaneous or gonadotropin-induced oocyte maturation, possibly by modulating the intracytoplasmic cAMP concentrations *via* a Ca^2+^-sensitive adenylate cyclase (96, 97).

In addition to the PLC–PKC–Ca^2+^ pathway, kisspeptin also induces other intracellular transduction pathways. The activation of ERK1/2 is thought to be the most conserved kinase signal among many cell types examined. However, not all cell types with the activation of ERK1/2 show stable p38 MAPK and PI3K/Akt activation when exposed to kisspeptin (98). In Chinese hamster ovary K1 cells, treatment with kisspeptin-10 induced a strong and sustained phosphorylation of ERK1/2, while a weak phosphorylation of p38 and no phosphorylation of stress-activated protein kinase/c-Jun NH_2_-terminal kinase (23). Similarly, kisspeptin can activate the ERK1/2 signaling without any effect on P38 signaling in rat luteal cells (37). Intriguingly, activated KISS1R can also recruit arrestin β-1 and β-2 to the plasma membrane, which further modulates the intracellular phosphorylated ERK1/2 levels in many mammalian cells (99–101). The attenuation of MAPK signaling pathway in GCs results in cell apoptosis and the subsequent follicle atresia (102). Interestingly, *Kiss1* or *Kiss1r* mutant animals showed much more atretic follicles than the wild-type counterparts (54, 55), suggesting that kisspeptin/KISS1R system may prevent the apoptosis of GCs. However, kisspeptin also downregulates the expression of FSHR in rat follicles, which may increase follicle atresia by inhibiting the follicular growth and inducing apoptosis of GCs (103, 104). Therefore, we may speculate that the intraovarian kisspeptin/KISS1R system modulates GC proliferation and apoptosis, oocyte maturation, ovulation, and steroidogenesis by regulating the MAPK signaling pathway.

Apart from the MAPK pathway, PI3K/Akt pathway also functions in both GCs and oocytes (105). In mammals, the PI3K/AKT signaling pathway is required for primordial follicle survival and activation, determination of the primordial follicle pool, and transition of the primordial follicle to growing follicles (106). In addition, this intra-follicular signaling modulates GC apoptosis, oocyte meiosis resumption, polar body emission, and spindle organization (107, 108). The effect of kisspeptin on the activation of PI3K/Akt signaling pathway is cell type specific. In rat luteal and thyroid cancer cells, kisspeptin cannot stimulate the phosphorylation of PI3K/Akt (37, 109), whereas kisspeptin induced the phosphorylation of PI3K/Akt in stably *KISS1R*-overexpressed thyroid cancer cells (110). Similarly, kisspeptin-10 was reported to inhibit the phosphorylation of PI3K/Akt in tumor cells (111–113), while kisspeptin-10 promoted the activation of PI3K/Akt in preoptic neurons (114). Whether the intraovarian kisspeptin/KISS1R system can modulate PI3K/Akt signaling pathway and further regulate GC apoptosis and oocyte maturation remains to be elucidated.

In HT-1080 cell line, overexpression with *KISS1* inhibited matrix metalloproteinase-9 (MMP-9) enzyme activity *via* blocking nuclear factor κB (NF-κB) nuclear translocation and subsequently reducing the capacity of NF-κB binding to the *MMP-9* promoter (115). This kisspeptin-induced signaling pathway has been demonstrated in several cancers, including urothelial carcinoma (116), ovarian epithelial cancers (117), and breast cancers (118). ERK and PI3K/Akt have been shown to act the upstream of NF-κB and regulate the NF-κB DNA-binding activity in melanoma cells (119–121). Interestingly, the enhanced expression of MMP-9 *via* PI3K/Akt/NF-κB (122) or ERK/NF-κB signaling pathway (123, 124) has also been established in other cell types. Therefore, it is most likely that ERK or PI3K/Akt signaling is involved in kisspeptin-induced downregulation of NF-κB/MMP-9.

Matrix metalloproteinase-9 is a matrix metalloproteinase that plays a critical role in tissue remodeling and follicular rupture (125). In addition, MMP-9 is involved in the mechanism by which kisspeptin prevents the tumor metastasis (112, 115, 126). In rats, intraovarian bursa administration of kisspeptin antagonist p234 resulted in the distortion of corpus luteum (14), indicating that kisspeptin can inhibit the degradation of the extracellular matrix in the ovary.

## Roles of the Kisspeptin/KISS1R System in Female Reproductive Pathology

Genetic analysis in humans gave the first evidence of the indispensable role of kisspeptin/KISS1R system in the control of reproduction. Using complementary genetic approaches, *KISS1R* has been identified as the causative gene responsible for the consanguineous families with idiopathic hypogonadotropic hypogonadism (26, 27). In addition, an inactivating mutation of *KISS1* gene has been reported as causative for idiopathic hypogonadotropic hypogonadism (127). The subsequent animal studies also confirmed that target depletion of either *Kiss1* or *Kiss1r* had similar phenotypes of the human condition (27, 54).

## Premature Ovarian Failure

A series of animal studies indicate a direct role of kisspeptin signaling in the ovary, and the defect of kisspeptin/KISS1R system precipitates a state of POF (or primary ovarian insufficiency). The haploinsufficient *Kiss1r* mice displayed a premature decline in ovulatory rate, progressive loss of oocytes, and antral follicles, reduced numbers of preantral follicles, and reduced fertility (52). In addition, the ovarian tissues of these precocious ovarian aging mice showed atrophic appearance without growing follicles and corpora lutea during their 48 weeks of ages (52). Furthermore, the phenotype is associated with a decreased expression level of ovarian *Kiss1r* mRNA. Notably, the failure of follicular development and ovulation due to the absent function of Kiss1r cannot be rescued by the replacement with gonadotropins (52). In line with these results, the loss of NTRK2 and Kiss1r receptor-mediated signaling in mouse oocytes caused POF (45). Collectively, these findings suggest a direct role of kisspeptin/KISS1r system in the ovary. Data generated from animal studies may provide a potential contribution to the evaluation or screening of isolated heterozygous mutations of KISS1R to POF in humans.

## Polycystic Ovary Syndrome

Polycystic ovary syndrome (PCOS) is a heterogenous endocrine disorder that affecting reproductive-aged women. This highly prevalent disease is characterized by hyperandrogenism, ovulatory dysfunction, and metabolic dysregulation (128). In women with PCOS, the classic neuroendocrine dysfunction leading to the ovarian phenotype includes increased LH pulsatility, decreased FSH secretion, and perturbed LH–FSH ratios, which could be due to the disrupted GnRH secretion (128). Since kisspeptin/KISS1R system is the upstream central controller for inducing GnRH (and LH) secretion, we may speculate that kisspeptin levels will be higher in women with PCOS. Indeed, a recent study showed that serum kisspeptin levels were significantly higher in women with PCOS and that serum levels of kisspeptin were negatively correlated with those of FSH (129). In line with this result, other studies demonstrated that higher serum levels of kisspeptin in women with PCOS (130, 131).

During the menstrual cycle, the increased LH pulsatility in PCOS persisted throughout the luteal phase, which resulted in the persistent stimulation of androgen production by ovarian theca cells (132). In addition, women with PCOS displayed metabolic alterations, which manifest insulin resistance and hyperinsulinemia (133). Interestingly, serum levels of kisspeptin in women with PCOS were positively correlated with those of testosterone and DHEAS (129). Studies in mice showed that administration of kisspeptin significantly increased the serum levels of testosterone (134). The metabolic dysregulation has been demonstrated to exert a suppressive effect on different levels of the gonadotropin axis in patients with PCOS (135) and an inhibitory effect on *Kiss1* mRNA expression in the hypothalamus of rats (136). Furthermore, ovary-derived kisspeptins have been shown to play a role in regulating the secretion of gonadotropins (137). All these findings provide the available, albeit indirect, evidence supporting a potential link between PCOS and the kisspeptin/KISS1R system.

## Endometriosis

Endometriosis is a common benign gynecologic disease defined as the ectopic presence of endometrial glandular epithelium and stroma outside the uterus (138). At present, the detailed pathogenesis of this disease remains unclear despite extensive research. Although a benign lesion, endometriosis shares several characteristics of malignancy, such as cell invasion, motility, and adhesion, which is a unique paradigm of benign metastasis (139). Several metastasis suppressor genes have been identified to suppress the metastasis at different steps of the metastatic cascade (140). *KISS1* was originally identified as a human metastasis suppressor gene that is able to suppress the metastasis of melanoma and breast cancer (22). A recent study showed that the expression of kisspeptin (also known as metastin) is significantly higher in the glandular endometrium of endometriosis lesions compared with the eutopic glandular endometrium, indicating that kisspeptin is potentially implicated in the pathogenesis and maintenance of endometriosis (141). In contrast, other study did not detect the expression of kisspeptin in any endometrial tissue obtained from women with endometriosis (142). The discrepancy could be attributed to differences in study design and experimental methods. Future studies will focus on investigating the relationship between kisspeptin and endometriosis and evaluating the potential clinical application of kisspeptin as a marker for early and minimally invasive detection of endometriosis.

## Future Directions and Clinical Applications

**Figure 3:**
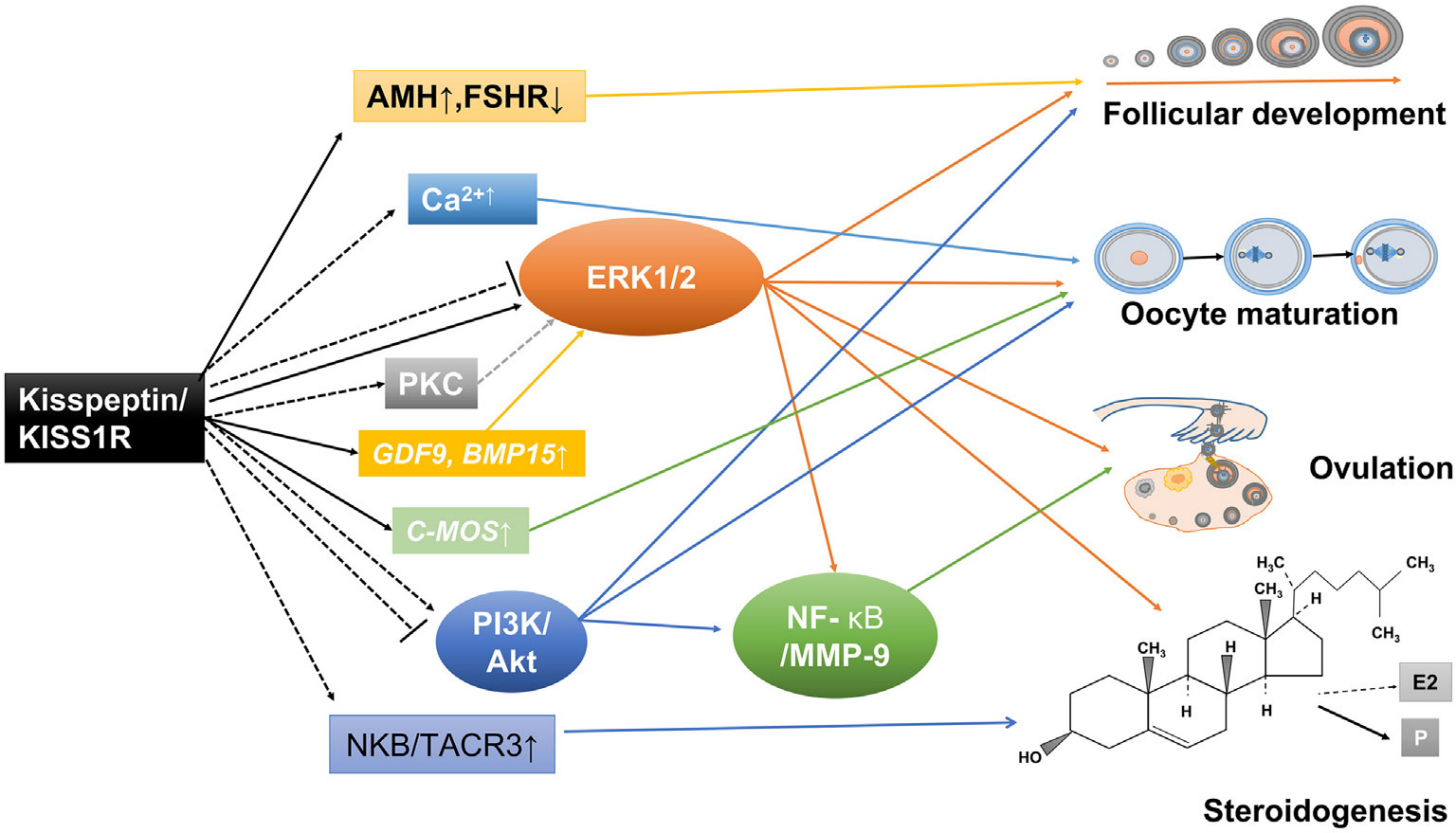
Potential mechanisms involved in the direct ovarian effects of the kisspeptin/kisspeptin receptor (KISS1R) system. Kisspeptin and KISS1R are expressed in ovarian cells. This locally produced kisspeptin might regulate follicular development, oocyte maturation, ovulation, and steroidogenesis in a paracrine or autocrine manner. Solid arrows stand for actions that have been clearly demonstrated in ovarian cells. Dotted arrows reflect potential pathways that could be involved in mediating the intraovarian kisspeptin/KISS1R effects, which have been proposed.

Although most research mainly focuses on the functional roles of the kisspeptin/KISS1R system in the central modulation of the H–P–G axis, the growing, albeit as yet limited, experimental data gathered recently suggest a putative role of kisspeptin signaling in the direct control of ovarian function (Figure 3). It must be stressed that our understanding of the physiological relevance, putative molecular mechanisms, and subsequent pathophysiological implications of such direct actions is still limited. However, such fragmentary evidence has supported the existence of a local kisspeptin/KISS1R system and the dysregulation of this system might contribute to several ovarian pathologies. Future study aimed at addressing the local role of kisspeptin in regulating ovarian function using a specific *Kiss1* or *kiss1r* knockout model in the ovary will be of great interest. Functional studies using the kisspeptin antagonist P234 or other inhibition approaches will help investigate the physiological and pathophysiological roles of kisspeptin in the ovarian biology (143, 144).

The involvement of kisspeptin/KISS1R system and its downstream signaling pathways in a range of ovarian function has led researchers to develop potential therapeutic approaches to overcome ovarian pathology and infertility. Data obtained from animal and human have indicated that the peripheral administration of kisspeptin-10 and kisspeptin-54 can initiate the LH surge (145, 146). At present, kisspeptin-54 has been used to trigger oocyte maturation effectively in women undergoing *in vitro* fertilization (IVF) (66). Moreover, kisspeptin-54 may be a substitute medication to trigger oocyte maturation during IVF treatment for women at high risk of developing ovarian hyperstimulation syndrome (67, 147, 148). However, safety concern should be seriously taken into consideration before its application, as kisspeptin supplementation was reported to have a harmful impact on the cultured hatched blastocysts in pig (44). Nevertheless, data from several clinical studies have shown that administration of kisspeptin in humans is safe without any observed adverse effects (149–151). More extensive clinical trials are required to investigate the safety, efficacy, and administration routes of the pharmaceutical applications of kisspeptins in humans. Animal studies have demonstrated that administration of pharmaceutical KISS1R antagonist suppresses the secretion of reproductive hormones (144), indicating a potential development of therapeutic targets for precocious puberty, endometriosis, some hormone-dependent cancers, and an alternative form of contraception.

During follicular development, kisspeptin suppressed the initial follicle recruitment through the upregulation of circulating AMH, which is able to inhibit the activation of primordial follicles (58). We may expect the clinical application of kisspeptin as a potential biomarker for ovarian reserve and an indicator for ovulation induction during IVF treatment. Studies in mice demonstrated that the haploinsufficient *Kiss1r* mice displayed a phenotype of POF (52). Such data will remind us a more careful evaluation of the possible attribution of certain heterozygous gene mutations in KISS1R to POF in humans and provide a useful screening method for these genetic variants.

## Conclusion

In the past decade, research regarding locally produced kisspeptin in the ovary has been of considerable interest. Emerging evidence indicates that the intraovarian kisspeptin/KISS1R system is of great importance in controlling female reproduction, including follicular development, oocyte maturation, steroidogenesis, and ovulation. Any abnormality or dysregulation of kisspeptin signaling may negatively affect the ovarian function, leading to reproductive pathology or female infertility. In this review, we provided a concise overview of the available, mainly indirect, evidence suggesting the local effects of the kisspeptin/KISS1R system in regulating ovarian function and the potential underlying molecular mechanisms. The conclusive demonstration of the physiological and pathophysiological roles of kisspeptin signaling in the ovary is still pending, and additional studies are required to better characterize the kisspeptin/KISS1R system in reproductive biology and pathology. Expanding our understanding of the expression, actions, and molecular mechanisms of this system in the human ovary is essential for determining whether therapeutic interventions targeting kisspeptin signaling can ameliorate several reproductive pathology and infertility.

## Author Contributions

K-LH collected the information, designed the pictures, wrote the manuscript, and manuscript submission. HZ collected the information and joined critical discussion. H-MC critically revised the manuscript and contributed to the conception of design. YY collected the information, designed the pictures, critically revised the manuscript, and contributed to the conception of design. JQ contributed to the conception of design and critical discussion.

## Conflict of Interest Statement

We declare that all authors have no conflict of interest with the contents of this manuscript.
